# A transformation perspective on marginal and conditional models

**DOI:** 10.1093/biostatistics/kxac048

**Published:** 2022-12-19

**Authors:** Luisa Barbanti, Torsten Hothorn

**Affiliations:** Institut für Epidemiologie, Biostatistik und Prävention, Universität Zürich, Hirschengraben 84, CH-8001 Zürich, Switzerland; Institut für Epidemiologie, Biostatistik und Prävention, Universität Zürich, Hirschengraben 84, CH-8001 Zürich, Switzerland

**Keywords:** Categorical data analysis, Conditional mixed models, Marginal models, Marginal predictive distributions, Survival analysis

## Abstract

Clustered observations are ubiquitous in controlled and observational studies and arise naturally in multicenter trials or longitudinal surveys. We present a novel model for the analysis of clustered observations where the marginal distributions are described by a linear transformation model and the correlations by a joint multivariate normal distribution. The joint model provides an analytic formula for the marginal distribution. Owing to the richness of transformation models, the techniques are applicable to any type of response variable, including bounded, skewed, binary, ordinal, or survival responses. We demonstrate how the common normal assumption for reaction times can be relaxed in the sleep deprivation benchmark data set and report marginal odds ratios for the notoriously difficult toe nail data. We furthermore discuss the analysis of two clinical trials aiming at the estimation of marginal treatment effects. In the first trial, pain was repeatedly assessed on a bounded visual analog scale and marginal proportional-odds models are presented. The second trial reported disease-free survival in rectal cancer patients, where the marginal hazard ratio from Weibull and Cox models is of special interest. An empirical evaluation compares the performance of the novel approach to general estimation equations for binary responses and to conditional mixed-effects models for continuous responses. An implementation is available in the tram add-on package to the R system and was benchmarked against established models in the literature.

## 1 Introduction

In the context of the analysis of dependent data or clustered observations, many statistical approaches for fitting conditional and marginal models have been studied. Generalized mixed-models (GLMMs, Stroup, 2012) condition on unobservable random effects and allow interpretation of covariate effects among subjects sharing the same value of such a random effect. Conditional models typically assume a specific random effects distribution and thus induce a joint distribution from which marginal distributions can be derived either analytically or by numerically integrating over random effects. Models formulating marginal covariate associations without requiring a model for the joint or conditional distribution can be estimated by solving generalized estimating equations (GEEs, Zeger *and others*, 1986, 1988). Later, marginalized multilevel models (Heagerty, 1999; Heagerty and Zeger, 2000) were introduced providing a likelihood-based approach to estimate marginal coefficients in the framework of a conditional model. Gory *and others* (2021) contribute a model definition allowing parameters estimated in a conditional model to be interpreted in a marginal fashion, and McGee and Stringer (2022) discuss marginal additive models for potentially nonlinear population-averaged associations, starting from a generalized additive mixed-model framework.

Marginal predictive distributions with interpretable parameters are easy to derive from normal linear mixed-effects models (LMMs) and binary probit GLMMs as well as from some frailty models using a copula representation (Goethals *and others*, 2008). Regression coefficients in other generalized linear mixed-effects or frailty models for non-normal responses (binary logistic, Poisson, or Cox normal frailty models, for example) only have a conditional interpretation, that is, given unobservable normal random effects. It is possible to obtain the marginal covariate effect by integrating out the normal random effects; however, the simple interpretability of the fixed-effects regression coefficients is then lost. In contrast, marginal models allowing a marginal interpretation of effects cannot be defined in an unambiguous way without the specification of a joint distribution and we refer to Lee and Nelder (2004) and Muff *and others* (2016) for a broader discussion of these issues.

Herein, we address the problem of formulating and estimating linear transformation models for the joint distribution of cluster-correlated observations arising, for example, in multicenter trials or when a subject is repeatedly examined over time. In contrast to many methods in the mixed-effects and frailty literature primarily aiming at explanation, that is, inference for regression coefficients conditional on random effects in the presence of correlated observations, the focus of this article is on inference for marginal distributions which can be derived analytically from this novel joint model.

Transformation models for correlated observations have been studied mostly in the survival analysis context. Parameter estimation is typically performed by nonparametric maximum likelihood estimation (Cai *and others*, 2000; Zeng *and others*, 2017) where the transformation function is only allowed to jump at distinct observed event times and is conceptually understood as an infinite-dimensional nuisance parameter. Lin *and others* (2017) even go a step further and propose a maximum rank correlation estimator for estimating the regression coefficients along with the mean of random effects while refraining from specifying neither the transformation function nor a distribution for the random effects. While this model is extremely general, the interpretation of the parameters is unclear and predictive distributions cannot be derived. Transformation models estimated in a fully parametric way (McLain and Ghosh, 2013; Hothorn *and others*, 2014, 2018; Klein *and others*, 2022) are practically as flexible as semiparametrically estimated models yet technically much easier to handle. Therefore, various flavors of conditional mixed-effects transformation models have been suggested (Manuguerra and Heller, 2010; Garcia *and others*, 2019; Tang *and others*, 2018; Sun and Ding, 2021; Tamási *and others*, 2022).

The results presented herein allow the formulation and estimation of models for the joint multivariate distribution of clustered non-normally distributed responses. The marginal distributions obtained from the joint distribution of observations in the same cluster feature directly interpretable marginal effects. Analytic formulae of the log-likelihood and the corresponding score function for absolute continuous and potentially non-normal responses observed without censoring are available. For censored and discrete observations, evaluation of the likelihood requires evaluation of multivariate normal probabilities; however, in low dimensions such that the approximation error can be made arbitrarily small in a reasonable time. Applications from different domains presented in Section 3 highlight that the methodology helps to unify models and inference procedures for clustered observations across traditionally compartmentalized subdisciplines of statistics. An empirical evaluation of marginal effects and distributions estimated by this novel approach is presented in Section 4.

## 2 Methods

Linear transformation models for the conditional distribution function

(2.1) $$P(Y\leq y|X=x)=F\left(h(y)-x^{⊤}\beta \right)$$

of some univariate and at least ordered response Y∈Ξ given a configuration ***x*** of covariates ***X*** are defined by three objects: An “inverse link function” *F*, a linear predictor $x^{⊤}\beta$ with regression coefficients β*excluding* an intercept, and a monotone nondecreasing transformation, or “intercept,” function *h*. Only β and *h* are unknowns to be estimated from data whereas *F* defines the scale linearity of the effects is assumed upon. This model class covers many prominent regression models, such as normal, log-normal, Weibull, or Cox models for absolute continuous responses, binary models with different link functions, proportional odds, and hazards cumulative models for ordered responses, and many less well-known or even novel models (Hothorn *and others*, 2018). A summary of the possible inverse link functions *F* and of the corresponding interpretation of the linear predictor $x^{⊤}\beta$ is given in Table 1. This flexible modeling framework covers probabilistic index models (Thas *and others*, 2012), that is, models allowing interpretation of the effect size in terms the probability that a randomly selected subject has an outcome greater than the one of another randomly selected subject, given that the covariate values for both subjects are known (for instance, whether a subject received treatment or not). We illustrate the usefulness of this quantity in Sections 3.3 and 3.4.

**Table 1:** *Interpretation of the linear predictor* $x^{⊤}\beta$*under different inverse link functions F*. *In practice, many models are known with respect to the link function* $F^{-1}$, *which we report accordingly. We denote the baseline* ($x^{⊤}\beta =0$) *cumulative distribution function by* F(h(y))*and the conditional cumulative distribution function by* F(h(y)|x)

$F^{-1}(z)$	*F*(*z*)	Interpretation of $x^{⊤}\beta$
probit	$\Phi_{0,1}(z)$	conditional mean
	Standard normal	$E(h(Y)|x)=x^{⊤}\beta$
logit	${\text{logit}}^{-1}(z)=\frac{1}{1+\text{exp}(-z)}$	log-odds ratio
	Standard logistic	$\frac{F(h(y)|x)}{1-F(h(y)|x)}=\text{exp}(-x^{⊤}\beta )\frac{F(h(y))}{1-F(h(y))}$
cloglog	cloglog${\;}^{-1}(z)=1-\text{exp}(-\text{exp}(z))$	log-hazard ratio
	Gompertz/Min. Extreme Value	$1-F(h(y)|x)={\left(1-F(h(y))\right)}^{\;\text{exp}(-x^{⊤}\beta )}$
loglog	loglog${\;}^{-1}(z)=\text{exp}(-\text{exp}(-z))$	log-reverse time hazard ratio
	Gumbel/Max. Extreme Value	$F(h(y)|x)=F{\left(h(y)\right)}^{\;\text{exp}(x^{⊤}\beta )}$

For clustered or longitudinal data, we observe multiple values of the response *Y* for each observational unit (clusters or subjects) whose interdependencies are not reflected in model (2.1). Adding, in analogy to GLMMs, a random effects term $u^{⊤}r$ to the linear predictor in (2.1) defines mixed-effects transformation models

(tramME) $$P_{Y}(Y\leq y|x,u,r)=F(h(y)-x^{⊤}\beta -u^{⊤}r).$$

When the random effects follow a specific bridge distribution to *F*, that is, the normal distribution for F=Φ, the stable distribution when $F={\text{cloglog}}^{-1}$ (Aalen *and others*, 2008), or the distribution derived by Wang and Louis (2003) for $F={\text{logit}}^{-1}$, marginal distributions can be derived. Neither the likelihood nor marginal distributions, and thus a marginal interpretation of β, are available in closed form when the model is formulated differently, especially when normal random effects are coupled with F≠Φ (Tamási *and others*, 2022).

### 2.1 Joint transformation models

To address these issues, we present a novel transformation model for the joint distribution that provides simple analytic expressions for marginal predictive distributions of the form (2.1). In this setup, i=1,…,N independent observational units, each consisting of *N_i_* correlated observations of the response $Y_{i}={\left(Y_{i1},\ldots ,Y_{iN_{i}}\right)}^{⊤}\in \Xi^{N_{i}}$, are available for estimating the joint distribution. While refraining to specify a certain parametric joint multivariate distribution for $Y_{i}$, we assume that probabilities on the scale of a suitable *transformation* of $Y_{i}$ can be evaluated using a multivariate normal distribution whose structured covariance matrix captures the correlations between the transformed elements of $Y_{i}$. The aim of this article is to simultaneously estimate the transformation, regression coefficients, and the structured covariance from data using models which emphasize predictive distributions and parameter interpretability.

The nondecreasing transformation function h:Ξ→R is applied element-wise to the response vector $h_{N_{i}}(Y_{i})={\left(h(Y_{i1}),\ldots ,h(Y_{iN_{i}})\right)}^{⊤}$ ensuring that the same transformation is applied to all *N_i_* observations. Together with $Y_{i}$, one observes a corresponding matrix $X_{i}={\left(x_{i1}|\ldots |x_{iN_{i}}\right)}^{⊤}\in R^{N_{i}\times Q}$ of full rank containing treatment assignment or covariates whose corresponding regression coefficients β are of interest. In addition, the design of the experiment is described by a matrix $U_{i}={\left(u_{i1}|\ldots |u_{iN_{i}}\right)}^{⊤}\in R^{N_{i}\times R}$. We exclusively study setups with simple cluster assignment encoded in this matrix ($U_{i}={\left(1\right)}_{N_{i},1}$) or longitudinal data ($u_{ij}=(1,t_{ij})$ indicating that *Y_ij_* for the *i*th subject was observed for at time *t_ij_*). We propose to study models for the joint distribution function of $Y_{i}$ given $X_{i}$ and $U_{i}$ of the form

(2.2) $$P(Y_{i}\leq y|X_{i},U_{i})=\Phi_{0_{N_{i}},\Sigma_{i}(\gamma )}\left(D_{i}(\gamma )\Phi_{N_{i}}^{-1}(F_{N_{i}}\{D_{i}{\left(\gamma \right)}^{-1}[h_{N_{i}}(y)-X_{i}\beta ]\})\right).$$

Here, $\Phi_{0_{N_{i}},\Sigma_{i}(\gamma )}\left(\cdot \right)$ is the distribution function of an *N_i_*-dimensional normal random vector with mean vector zero and structured covariance matrix

$$\Sigma_{i}(\gamma ):=U_{i}\Lambda (\gamma )\Lambda {\left(\gamma \right)}^{⊤}U_{i}^{⊤}+I_{N_{i}}$$

as defined by the random effects design matrix and an unstructured Cholesky factor $\Lambda (\gamma )\in R^{R\times R}$ depending on unknown variance parameters $\gamma \in R^{R(R+1)/2}$; $I_{N_{i}}$ denotes the $N_{i}\times N_{i}$ identity matrix. We isolate the square roots of the diagonal elements of $\Sigma_{i}(\gamma )$ in the matrix $D_{i}(\gamma )=\text{diag}{\left(\Sigma_{i}(\gamma )\right)}^{1/2}\cdot I_{N_{i}}=\text{diag}{\left(U_{i}\Lambda (\gamma )\Lambda {\left(\gamma \right)}^{⊤}U_{i}^{⊤}+I_{N_{i}}\right)}^{1/2}\cdot I_{N_{i}}$. A positive-semidefinite covariance matrix $\Sigma_{i}(\gamma )$ is given under the constraint $\text{diag}(\Lambda (\gamma ))\geq 0_{R}$. For the simple model with $U_{i}={\left(1,\ldots ,1\right)}^{⊤}$, we have $\Lambda (\gamma )=\gamma_{1},\;\Sigma_{i}(\gamma )={\left(\gamma_{1}^{2}\right)}_{N_{i},N_{i}}+I_{N_{i}}$, and $D_{i}{\left(\gamma \right)}^{-1}={\left(\gamma_{1}^{2}+1\right)}^{-1}\cdot I_{N_{i}}$ is a scaling factor to the transformation function *h* and regression coefficients β which is instrumental for the derivation of marginal distributions. In the longitudinal setup, $\Lambda =\left(\begin{array}{cc} \gamma_{1} & 0\\ \gamma_{2} & \gamma_{3} \end{array}\right)$ and the covariance $\Sigma_{i}{\left(\gamma \right)}_{j,ȷ}$ depends on the observation times *t_ij_* and $t_{iȷ}$. The key component is the shifted transformation $h_{N_{i}}(y)-X_{i}\beta$ modeling the impact of the regression coefficients on the transformed scale.

The transformation function *h*, the regression coefficients β, and the variance parameters γ are unknowns to be estimated from data. In (2.2), $\Phi_{N_{i}}^{-1}(p)={\left(\Phi^{-1}(p_{1}),\ldots ,\Phi^{-1}(p_{N_{i}})\right)}^{⊤}$ applies the quantile function $\Phi^{-1}$ of the standard normal element-wise to some vector of probabilities $p={\left(p_{1},\ldots ,p_{N_{i}}\right)}^{⊤}\in {\left(0,1\right)}^{N_{i}}$. Furthermore, F:R→[0,1] is an *a priori* defined cumulative distribution function of some absolute continuous distribution with log-concave density *f*; $F_{N_{i}}$ and $f_{N_{i}}$ are the element-wise applications of *F* and *f*, respectively.

**Fig. 1:**
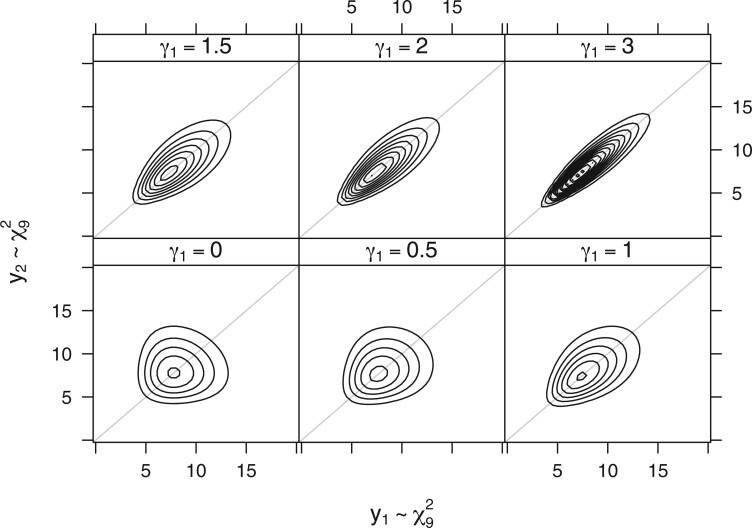
Illustration. Bivariate joint density of an unconditional logistic ($F=l\mathrm{ogi}t^{-1}$) transformation model for repeated measures (cluster size $N_{i}\equiv 2,\;U_{i}={\left(1,1\right)}^{⊤}$ and $\Sigma_{i}=\gamma_{1}^{2}U_{i}U_{i}^{⊤}+I_{2}$) with transformation functions $h_{1}=h_{2}=\sqrt{1+\gamma_{1}^{2}}\cdot l\mathrm{ogit}{}^{\circ}\chi_{9}^{2}$ such that both marginal distributions follow the $\chi_{9}^{2}$ law. For $\gamma_{1}=0$, observations within a cluster are independent, and their correlation increases with increasing values of *γ*_1_.

For absolute continuous responses $Y_{i}\in R^{N_{i}}$, model (2.2) implies that the latent variable

(2.3) $$Z_{i}:=D_{i}(\gamma )\Phi_{N_{i}}^{-1}(F_{N_{i}}\{D_{i}{\left(\gamma \right)}^{-1}[h_{N_{i}}(Y_{i})-X_{i}\beta ]\})\in R^{N_{i}}$$

defined as an element-wise transformation of the observations $Y_{i}$ follows a multivariate normal distribution $Z_{i}∼N_{N_{i}}\left(0_{N_{i}},\Sigma_{i}(\gamma )\right)$. The model is distribution-free in the sense that for a baseline configuration (with $X_{i}\beta =0_{N_{i}}$), such a transformation into multivariate normality exists for all marginal distributions (Klein *and others*, 2022). The model does, however, impose a certain correlation structure through the choice of $U_{i}$. An example for the joint distributions induced by increasing correlations among bivariate repeated measurements with skewed marginal distributions is given in Figure 1.

The key aspect of an implementation of model (2.2) is the parameterization of the transformation function as $h_{N_{i}}(y)=A(y)\vartheta ,$ where $A(y)={\left(a(y_{1})|\ldots |a(y_{N_{i}})\right)}^{⊤}\in R^{N_{i}\times P}$ is the matrix of evaluated basis functions $a:\Xi \to R^{P}$. Choices of basis functions ***a*** are problem-specific and several options are discussed in Section 3 and, in more detail, in Hothorn *and others* (2018) and Hothorn (2020).

### 2.2 Connection to normal LMMs

We first consider the special case F=Φ, where the transformation of $Y_{i}$ simplifies to $Z_{i}=h_{N_{i}}(Y_{i})-X_{i}\beta =A(Y_{i})\vartheta -X_{i}\beta$. Model (2.2) contains the LMM as a special case. In its standard notation, the LMM reads

(LMM) $$Y_{i}=\alpha +X_{i}\tilde{\beta }+U_{i}R_{i}+\sigma \varepsilon_{i}$$

with random effects $R_{i}∼N_{R}(0_{R},G(\gamma ))$, residuals $\varepsilon_{i}∼N_{N_{i}}(0_{N_{i}},I_{N_{i}})$ under the assumption $R_{i}⊥\varepsilon_{i}$, intercept α∈R and residual standard deviation $\sigma \in R^{+}$. The matrices $X_{i}$ and $U_{i}$ are typically referred to as “fixed effects” and “random effects” design matrices in the literature. This model can be reformulated as a model for the joint multivariate distribution

(2.4) $$Z_{i}=\frac{Y_{i}-\alpha -X_{i}\tilde{\beta }}{\sigma }=U_{i}\sigma^{-1}R_{i}+\varepsilon_{i}∼N_{N_{i}}\left(0_{N_{i}},U_{i}\Lambda (\gamma )\Lambda {\left(\gamma \right)}^{⊤}U_{i}^{⊤}+I_{N_{i}}\right)$$

based on the relative covariance factorization $\sigma^{-2}G(\gamma )=\Lambda (\gamma )\Lambda {\left(\gamma \right)}^{⊤}\in R^{R\times R}$. This is model (2.2) with F=Φ, linear transformation $h_{N_{i}}(Y_{i})={\left(\sigma^{-1}(Y_{i1}-\alpha ),\ldots ,\sigma^{-1}(Y_{iN_{i}}-\alpha )\right)}^{⊤}=A(Y_{i})\vartheta$ with linear basis functions $a(y)={\left(y,-1\right)}^{⊤}$ and parameters $\vartheta ={\left(\sigma^{-1},\alpha \sigma^{-1}\right)}^{⊤}$, and finally fixed effects $\beta =\sigma^{-1}\tilde{\beta }$.

Using this notation, the conditional distribution function of some element Y∈Ξ of ***Y***, conditional on ***x***, ***u***, and unobservable random effects R=r, is

$$P(Y\leq y|x,u,r)=\Phi \left(a{\left(y\right)}^{⊤}\vartheta -x^{⊤}\beta -\sigma^{-1}u^{⊤}r\right).$$

The marginal distribution of some element Y∈Ξ of ***Y***, which is still conditional on ***x*** and ***u*** but integrates over the random effects ***R***, can be obtained from the joint multivariate normal (2.4) as

$$P(Y\leq y|x,u)=\Phi \left(\frac{a{\left(y\right)}^{⊤}\vartheta -x^{⊤}\beta }{\sqrt{u^{⊤}\Lambda (\gamma )\Lambda {\left(\gamma \right)}^{⊤}u+1}}\right).$$

The shrunken marginal fixed effects $\beta /\sqrt{u^{⊤}\Lambda (\gamma )\Lambda {\left(\gamma \right)}^{⊤}u+1}$ were also described by Wu and Wang (2019) in a Bayesian implementation of this model. Understanding the LMM as special case of a transformation model allows to relax the normality assumption for $Y_{i}$ by introducing nonlinear transformation functions $h(y)=a{\left(y\right)}^{⊤}\vartheta$ defined by a nonlinear basis ***a*** (Hothorn *and others*, 2018). Section 3.1 contains a comparison of the two models. Probit GLMMs for binary responses Y∈Ξ={0,1} can also be understood as a special case of a transformation model with intercept h(0)=α and h(1)=∞. Several implementations of such GLMMs are compared empirically to an implementation motivated from a transformation model perspective in Section 3.2.

### 2.3 Distinction from generalized mixed-effects and frailty models

Two important extensions of the LMM include GLMMs and frailty models. For binary responses, the logistic GLMM has the conditional, given normal random effects ***r***, interpretation

$$P(Y=0|x,u,r)={\text{logit}}^{-1}\left(\alpha +x^{⊤}\beta +u^{⊤}r\right).$$

In survival analysis with $Y\in \Xi =R^{+}$, a Weibull normal frailty model leads to the conditional interpretation

$$P(Y\leq y|x,u,r)={\text{cloglog}}^{-1}\left(\alpha_{1}+\alpha_{2}\;\text{log}(y)+x^{⊤}\beta +u^{⊤}r\right).$$

A normal frailty Cox model

$$P(Y\leq y|x,u,r)={\text{cloglog}}^{-1}\left(h(y)+x^{⊤}\beta +u^{⊤}r\right).$$

replaces the log-linear transformation function of the Weibull model with a smooth log-cumulative hazard function *h*(*y*). All three models are special cases of mixed-effects transformation models (tramME).

Assuming normal random effects ***u***, neither model can be understood in terms of model (2.2) and two main difficulties are associated with these types of models assuming additivity of the fixed and random effects on the log-odds ratio or log-hazard ratio scales. First, unlike in (LMM), there is no analytic expression for the marginal distribution and thus a marginal interpretation of the fixed effects β is difficult. Second, evaluation of the likelihood typically relies on a Laplace approximation of the integral with respect to the random effects’ distribution and problems with this approximation have been reported, for example by Ogden (2015). The novel multivariate transformation model for clustered observations based on (2.2) addresses both of these issues as shall be explained in the next subsections.

### 2.4 Transformation models with marginal interpretation

Simple analytic expressions for the marginal distribution are available (also for F≠Φ), independent of the choice of the basis function ***a***, noting that the variance of the *j*th element of $Z_{i}$ (2.3) is $u_{ij}^{⊤}\Lambda (\gamma )\Lambda {\left(\gamma \right)}^{⊤}u_{ij}+1$.

The Gaussian copula distribution of 2.2 directly implies the marginal distribution function in form of a marginal transformation model (mtram):

(mtram) $$\begin{array}{c} P(Y\leq y|x,u)=\Phi \left(\frac{\sqrt{u^{⊤}\Lambda (\gamma )\Lambda {\left(\gamma \right)}^{⊤}u+1}\Phi^{-1}\left(F\left(\frac{a{\left(y\right)}^{⊤}\vartheta -x^{⊤}\beta }{\sqrt{u^{⊤}\Lambda (\gamma )\Lambda {\left(\gamma \right)}^{⊤}u+1}}\right)\right)}{\sqrt{u^{⊤}\Lambda (\gamma )\Lambda {\left(\gamma \right)}^{⊤}u+1}}\right)\\ =F\left(\frac{a{\left(y\right)}^{⊤}\vartheta -x^{⊤}\beta }{\sqrt{u^{⊤}\Lambda (\gamma )\Lambda {\left(\gamma \right)}^{⊤}u+1}}\right). \end{array}$$

In this model, the fixed effects β divided by $\sqrt{u^{⊤}\Lambda (\gamma )\Lambda {\left(\gamma \right)}^{⊤}u+1}$ are directly interpretable given U=u, for example as log-odds ratios ($F={\text{logit}}^{-1}$) or log-hazard ratios ($F={\text{cloglog}}^{-1}$). Because $\Lambda (\gamma )\Lambda {\left(\gamma \right)}^{⊤}$ is positive semidefinite, there might be a reduction in effect size when comparing the fixed effects β from formula (2.2) to the marginal effects $\beta /\sqrt{u^{⊤}\Lambda (\gamma )\Lambda {\left(\gamma \right)}^{⊤}u+1}$ from model (mtram). For repeated measurements with u=1 we get a constant reduction by $1/\sqrt{\gamma_{1}^{2}+1}$. In longitudinal models, the marginal effect at time *t* is $\beta /\sqrt{\gamma_{1}^{2}+\gamma_{1}\gamma_{2}t+(\gamma_{2}^{2}+\gamma_{3}^{2})t^{2}+1}$ because $u={\left(1,t\right)}^{⊤}$. For positively correlated random intercepts and random slopes (i.e., $\gamma_{2}>0$), the marginal effect always decreases over time.

### 2.5 The likelihood function

For parameters ϑ,β, and γ, the log-likelihood contribution $\ell_{i}(\vartheta ,\beta ,\gamma )$ of the *i*th subject or cluster is based on the transformation

(2.5) $$z(y|\vartheta ,\beta ,\gamma )=D_{i}(\gamma )\Phi_{N_{i}}^{-1}(F_{N_{i}}\{D_{i}{\left(\gamma \right)}^{-1}[A(y)\vartheta -X_{i}\beta ]\})$$

of some $y\in \Xi^{N_{i}}$.

For discrete or interval-censored observations $({\underline{y}}_{i},{\bar{y}}_{i}]\subset R^{N_{i}}$, the log-likelihood contribution is

(2.6) $$\begin{array}{c} \ell_{i}(\vartheta ,\beta ,\gamma )=\text{log}\;P\left({\underline{y}}_{i}\leq Y_{i}<{\bar{y}}_{i}\right)=\text{log}\;P\left(z({\underline{y}}_{i}|\vartheta ,\beta ,\gamma )\leq Z_{i}<z({\bar{y}}_{i}|\vartheta ,\beta ,\gamma )\right)\\ =\text{log}\;\left\{\Phi_{0_{N_{i}},\Sigma_{i}(\gamma )}\left[z({\underline{y}}_{i}|\vartheta ,\beta ,\gamma ),z({\bar{y}}_{i}|\vartheta ,\beta ,\gamma )\right]\right\}, \end{array}$$

where

$$\Phi_{0_{N_{i}},\Sigma_{i}(\gamma )}(\underline{z},\bar{z})=\int_{\underline{z}}^{\bar{z}}\phi_{0_{N_{i}},\Sigma_{i}(\gamma )}(z)dz$$

is the integral over the *N_i_*-dimensional multivariate normal density $\phi_{N_{i}}$ with mean zero and covariance $\Sigma_{i}$. The structure of $\Sigma_{i}(\gamma )$ can be exploited to dramatically reduce the dimensionality of the integration problem. Applying the procedure by Marsaglia (1963), one can reduce this *N_i_*-dimensional integral to an *R*-dimensional integral over the unit cube (see Appendix A).

For continuous observations $y\in R^{N_{i}}$, it is common practice (Section 5, Lindsey, 1999) to approximate this log-likelihood by a log-density evaluated at the observations $y_{i}$:

(2.7) $$\begin{array}{c} \ell_{i}(\vartheta ,\beta ,\gamma )\approx -\frac{1}{2}\text{log}\;\left|\Sigma_{i}(\gamma )\right|+\\ -\frac{1}{2}z{\left(y_{i}|\vartheta ,\beta ,\gamma \right)}^{⊤}(\Sigma_{i}{\left(\gamma \right)}^{-1}-D_{i}{\left(\gamma \right)}^{-2})z(y_{i}|\vartheta ,\beta ,\gamma )+\\ \;\;{\text{log}}_{N_{i}}{\left(f_{N_{i}}\{D_{i}{\left(\gamma \right)}^{-1}[A(y_{i})\vartheta -X_{i}\beta ]\}\right)}^{⊤}1_{N_{i}}+\\ \;\;{\text{log}}_{N_{i}}{\left(A\prime (y_{i})\vartheta \right)}^{⊤}1_{N_{i}}, \end{array}$$

where the Cholesky factorization $L_{i}(\gamma )L_{i}{\left(\gamma \right)}^{⊤}=\Sigma_{i}(\gamma )$ is utilized. It should be noted that the exact log-likelihood function (2.6) does not require the precision matrix $\Sigma_{i}{\left(\gamma \right)}^{-1}$ to be computed. In the above approximation, ${\;\text{log}\;}_{N_{i}}$ is the element-wise natural logarithm and $f_{N_{i}}$ the element-wise density of *F*. $A\prime (y_{i})$ denotes the matrix of evaluated derivatives a′ of the basis function ***a***. The log-likelihood of 2.7 is derived in Appendix B.

Using either log-likelihood, we obtain simultaneous maximum-likelihood estimates for all model parameters from

$$({\hat{\vartheta }}_{N},{\hat{\beta }}_{N},{\hat{\gamma }}_{N})=\underset{(\vartheta ,\beta ,\gamma )\in R^{P+Q+M}}{\text{argmax}}\sum_{i=1}^{N}\ell_{i}(\vartheta ,\beta ,\gamma ).$$

Some models require additional constraints on ϑ to be implemented (Hothorn *and others*, 2018). Analytic score functions for all model parameters ϑ,β, and γ are available (see Appendix B). Score functions for the discrete or censored likelihood (2.6) and the observed Fisher information matrices for both likelihoods are obtained numerically. The full parameterization of *h* allows application of standard results for likelihood asymptotics (van der Vaart, 1998) to independent observations (Hothorn *and others*, 2018). Model (2.2) is a special case of the multivariate transformation model of Klein *and others* (2022) where the transformation function *h* and the fixed effects β are constrained to be the same for all “coordinates” of the random vector $Y_{i}$ (i.e., observations in the same cluster). Therefore, model (2.2) benefits from the same asymptotic results reported by Klein *and others* (2022).

## 3 Applications

In this section, we discuss four potential applications of marginally interpretable transformation models. Data, numerical details, and code reproducing the results are available from the Online Appendix (Barbanti and Hothorn, 2022). We start with two head-to-head comparisons where model (mtram) suggested here can be estimated by already existing software implementations of mixed-effects probit models for the purpose of validating the implementation of model (mtram) in the add-on package **tram** (Hothorn *and others*, 2022) to the R system for statistical computing.

### 3.1 Non-normal mixed-effects models

The average reaction times to a specific task over several days of sleep deprivation are given for i=1,…,N=18 subjects (Belenky *and others*, 2003). The data are often used to illustrate LMMs with correlated random intercepts and slopes of the form (LMM)

(3.8) $$P(\text{Reaction time}\leq y|\text{day},i)=\Phi \left(\frac{y-\alpha -\beta \text{day}-\alpha_{i}-\beta_{i}\text{day}}{\sigma }\right),(\alpha_{i},\beta_{i})∼N_{2}(0,G(\gamma )).$$

This conditional normal model can be estimated by maximizing the corresponding normal log-likelihood and distinct implementations of classical normal linear mixed models (LMM, package **lme4**, Bates *and others*, 2015), conditional mixed-effects transformation models (tramME, package **tramME**, Tamási and Hothorn, 2021), and marginal transformation models (mtram, package **tram**, Hothorn *and others*, 2022) provide identical results (in-sample log-likelihood –875.97).

Because the reaction times can hardly be expected to follow a symmetric distribution, we consider the non-normal conditional and marginal transformation model

(3.9) $$P(\text{Reaction}\leq y|\text{day},i)=\Phi \left(h(y)-\beta \text{day}-\alpha_{i}-\beta_{i}\text{day}\right),(\alpha_{i},\beta_{i})∼N_{2}(0,G(\gamma )),$$

where a monotonically increasing transformation function *h*(*y*) is allowed to deviate from linearity. Such probit-type mixed-effects models have been studied before, for example, by merging a Box–Cox power transformation *h* with a grid-search over REML estimates (Gurka *and others*, 2006), a conditional likelihood (Hutmacher *and others*, 2011), or a grid-search maximizing the profile likelihood (Maruo *and others*, 2017). Tang *and others* (2018) and Wu and Wang (2019) proposed a monotone spline parameterization of *h* in a Bayesian context.

We parameterize $h(y)=a{\left(y\right)}^{⊤}\vartheta$ in terms of a monotonically increasing polynomial in Bernstein form of order six (Hothorn *and others*, 2018). The conditional transformation model (Tamási *and others*, 2022) can be estimated by maximizing a Laplace approximation to the log-likelihood (Tamási and Hothorn, 2021) simultaneously with respect to all parameters ϑ, β, and γ. Direct optimization of the log-likelihood (2.7) for (mtram) leads to identical results (log-likelihood –859.55), because the conditional and marginal models are identical for F=Φ and the Laplace approximation is very accurate in this case. For F≠Φ, conditional and marginal transformation models differ, and numerical integration with respect to the normal random effects is required when marginal distributions shall be obtained from a conditional model. In contrast, the (mtram) provides a closed-form expression for marginal distributions for all choices of *F*. With $F={\text{logit}}^{-1}$, the log-likelihood of the marginal model increases slightly (–860.6377).

**Fig. 2:**
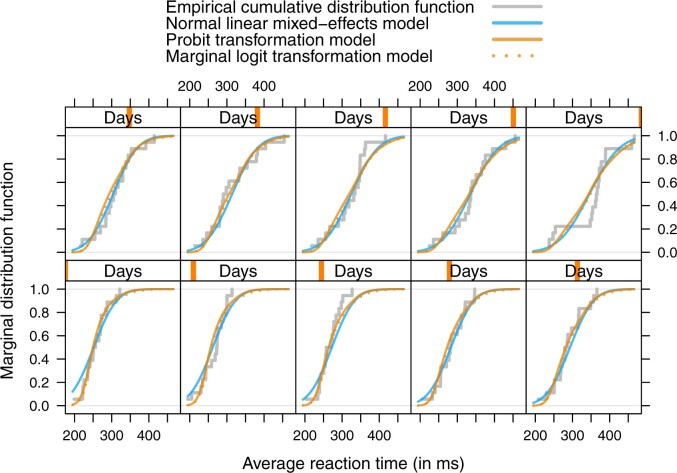
Sleep deprivation. Marginal distribution of reaction times, separately for each day of study participation. The grey step-function corresponds to the empirical cumulative distribution function, the blue line to the marginal cumulative distribution of the normal LMM (3.8), estimated by the lmer function from package **lme4** (Bates *and others*, 2015), the solid yellowish line to the probit transformation model (3.9), and the dotted yellowish line to the logistic marginal transformation model.

The daily marginal distribution functions of normal and non-normal models are compared to the daily marginal empirical cumulative distributions in Figure 2. Especially for short reaction times early in the experiment, the non-normal transformation models seem to fit the data better than the normal linear model. Between the probit transformation model and the logistic marginal transformation model, only minor discrepancies can be observed.

### 3.2 Binary marginal models

For a binary response Y∈{0,1}, the transformation h(y)=α reduces to a scalar intercept. Thus, maximization of the discrete log-likelihood (2.6) provides an alternative to commonly applied approximations, such as Laplace or Adaptive Gauss–Hermite Quadrature, for fitting conditional probit mixed-effects models. In addition, the possibility to interpret parameters marginally also for F≠Φ asks for a comparison to generalized estimation equations (GEEs).

We first compared different implementations of binary probit mixed-effects models for the notoriously difficult to handle toe nail data (Backer *and others*, 1998) for which quasi-separation issues have been reported (Sauter and Held, 2016). The ordinal response measuring toe nail infection was categorized to two levels. We were interested in binary probit models featuring fixed main and interaction effects *β*_1_, *β*_2_, and *β*_3_ of treatment (itraconazole vs. terbinafine) and time. Subject-specific random intercept models and models featuring correlated random intercepts and slopes were estimated by the glmer function from package **lme4** (Bates *and others*, 2015), by the glmmTMB function from package **glmmTMB** (Brooks *and others*, 2017), and by direct maximization of the exact discrete log-likelihood (2.6) given in Appendix A.

The estimated model parameters, along with the discrete log-likelihood (2.6) evaluated at these parameters, are given in Table 2. For the random intercept models, AGQ, the Laplace approximation in **glmmTMB**, and the discrete log-likelihood gave the same results, the Laplace approximation implemented in package **lme4** seemed to fail. It was not possible to apply the AGQ approach to the random intercept/random slope model. The two implementations of the Laplace approximation in packages **lme4** and **glmmTMB** differed for the random intercept but not for the random intercept/random slope model. The log-likelihood obtained by direct maximization of (2.6) resulted in the best fitting model with the least extreme parameter estimates. Computing times for all procedures were comparable.

**Table 2:** *Toe nail data. Binary probit models featuring fixed interceptsα*, *treatment effects β*_1_, *time effects β*_2_, *and time-treatment interactions β*_3_*are compared. Random intercept (RI) and random intercept/random slope (RI + RS) models were estimated by the Laplace (L) and Adaptive Gauss-Hermite Quadrature (AGQ) approximations to the likelihood (implemented in packages* ***lme4****and* ***glmmTMB****). In addition, the exact discrete log-likelihood (2.6) was used for model fitting and evaluation (the in-sample log-likelihood (2.6) for all models and timings of all procedures are given in the last two lines)*

	RI				RI + RS		
	glmer	glmer	glmmTMB		glmer	glmmTMB	
	L	AGQ	L	(2.6)	L	L	(2.6)
*α*	–3.39	–0.91	–1.10	0.91	–4.30	–4.30	1.58
*β*_1_	–0.03	–0.11	–0.17	–0.11	0.05	0.05	0.27
*β*_2_	–0.22	–0.19	–0.19	–0.19	–0.07	–0.07	–0.53
*β*_3_	–0.07	–0.06	–0.06	–0.06	–0.23	–0.23	–0.18
*γ*_1_	4.57	2.12	2.10	2.11	10.88	11.01	5.22
*γ*_2_	0.00	0.00	0.00	0.00	–1.64	–1.68	–0.37
*γ*_3_	0.00	0.00	0.00	0.00	0.79	0.83	0.53
LogLik	–675.22	–637.34	–638.54	–637.34	–628.12	–630.65	–545.12
Time (s)	3.83	2.40	2.04	2.20	7.53	3.44	8.08

In a second step, (mtram) with logit link was compared to marginal odds ratios obtained from a GEE. We refitted published GEE models for this data (SAS results in Chapter 10, Molenberghs and Verbeke, 2005) and noticed substantial differences indicating numerical instabilities for this data set (see Online Appendix, Barbanti and Hothorn, 2022). The monthly multiplicative treatment effect on the odds ratio scale was 0.91 (95% confidence interval 0.83–1.00) when a logistic GEE with unstructured working correlation was estimated. The logistic transformation model estimated the same parameter as 0.94 (95% confidence interval 0.89–0.99). Molenberghs and Verbeke (2005, p. 211) reported a GEE-based marginal odds ratio of 0.89 (95% confidence interval 0.81–0.98, with model-based standard errors and exp -transformed Wald intervals). The performance of GEEs and marginal transformation models are compared against ground truth in a simulation experiment in Section 4.

### 3.3 Models for bounded responses

**Fig. 3:**
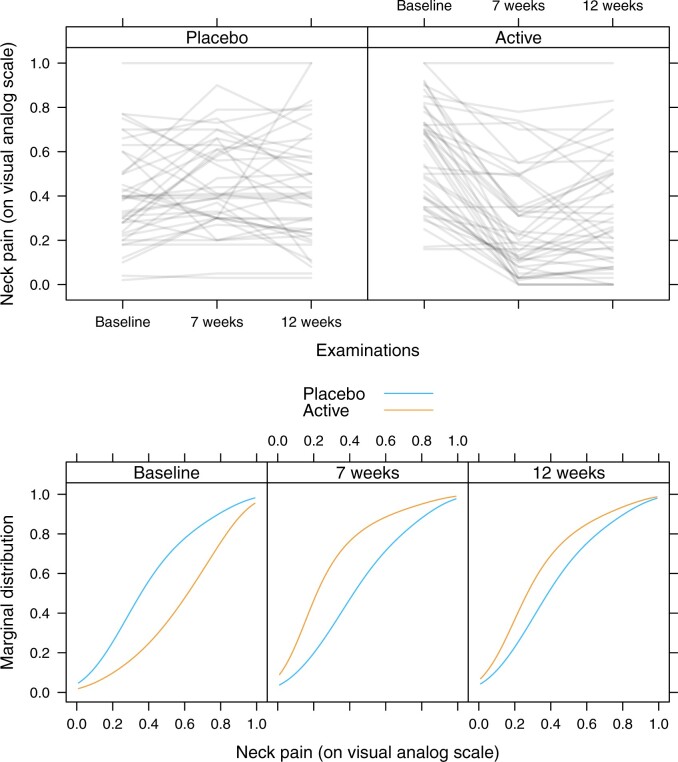
Neck pain. Pain trajectories of 90 subjects under active treatment or placebo evaluated at baseline, after 7 and 12 weeks (top) and marginal distribution functions of neck pain at the three different time points (bottom). These results were obtained from model (mtram) using $F={\text{logit}}^{-1}$ and a polynomial in Bernstein form *h*(*y*) on the unit interval.

Chow *and others* (2006) report on a randomized two-arm clinical trial comparing a novel neck pain treatment to placebo. Neck pain levels of 90 subjects were assessed at baseline, after 7, and after 12 weeks (complete trajectories are available for 84 subjects) on a visual analog scale. Manuguerra and Heller (2010) proposed a mixed-effects model for such a bounded response. The fixed effects are interpretable as log-odds ratios, conditional on random effects. The data are presented in the top panel of Figure 3. A transformation model (mtram) with $F={\text{logit}}^{-1}$ featuring a transformation function $h(y)=a{\left(y\right)}^{⊤}\vartheta$ defined by a polynomial in Bernstein form of order six on the unit interval, and correlated random intercept and random slope terms (u=(1,t) for times *t* = 0, 7, 12 weeks) is visualized by means of the corresponding marginal distribution functions in the bottom panel of Figure 3. Similar to the results reported earlier (Manuguerra and Heller, 2010), the model highlights more severe pain in the active treatment group at baseline. A positive treatment effect can be inferred after 7 weeks which seemed to level-off when subjects were examined after 12 weeks. It is important to note that these results have a marginal interpretation and that the model does not assume a specific distribution of the response, such as a Beta distribution for example.

From the marginally interpretable transformation models, relevant quantities, like the probabilistic index, can be derived (Online Appendix, Barbanti and Hothorn, 2022). In this application, the marginal probabilistic index is the probability that, for a randomly selected patient in the treatment group, the neck pain score at time *t* is higher than the score for a subject in the placebo group randomly selected at the same time point. We obtain a probability of 0.72 (95% confidence interval [0.58–0.83]) at baseline, 0.29 (95% confidence interval [0.17–0.43]) after 7 weeks, and 0.38 (95% confidence interval [0.24–0.54]) after 12 weeks.

### 3.4 Marginally interpretable survival models

The CAO/ARO/AIO-04 randomized clinical trial (Rödel *and others*, 2015) compared Oxaliplatin added to fluorouracil-based preoperative chemoradiotherapy and postoperative chemotherapy for rectal cancer patients to the same therapy using fluorouracil only. Patients were randomized in the two treatment arms by block randomization taking the study center, the lymph node involvement (negative vs. positive), and tumor grading (T1–3 vs. T4) into account. The primary endpoint was disease-free survival, defined as the time between randomization and nonradical surgery of the primary tumor (R2 resection), locoregional recurrence after R0/1 resection, metastatic disease or progression, or death from any cause, whichever occurred first. The observed responses are a mix of exact dates (time to death or incomplete removal of the primary tumor), right-censoring (end of follow-up or drop-out), and interval-censoring (local or distant metastases). The conditional hazard ratio 0.79 (0.64–0.98) was reported as obtained from a Cox mixed-effects model with normal random intercepts and without stratification fitted to right-censored survival times (Rödel *and others*, 2015). This means that a rectal cancer patient treated with the novel combination therapy benefits from a 21% risk reduction compared to a patient *from the same block* treated with fluorouracil only.

We were interested in estimating a marginally interpretable treatment effect (acknowledging the fact that patients enrolled into the trial were *not* a random sample from all rectal cancer patients) based on a marginally interpretable stratified (with respect to lymph node involvement and tumor grading) Weibull model for clustered observations (blocks) in the presence of interval-censored survival times. This model can be formulated by (mtram) choosing $F={\text{cloglog}}^{-1},\;a(y)={\left(1,\;\text{log}(y)\right)}^{⊤},\;u=1$ being the block indicator, and variance parameter *γ*_1_ (corresponding to the correlation structure of a random intercept only model) as well as a treatment parameter *β* (comparing the novum to fluorouracil only). Stratification was implemented by strata-specific parameters ϑ for each of the four strata. It should be noted that this model is not equivalent to a classical Weibull normal frailty model.

A confidence interval for the marginal hazard ratio $\text{exp}(\beta /\sqrt{\gamma_{1}^{2}+1})$ was computed by simulating from the joint normal distribution of $\left(\hat{\beta },{\hat{\gamma }}_{1}\right)$. With a relatively small ${\hat{\gamma }}_{1}=0.15$ (with standard error 0.13), this resulted in a marginal hazard ratio of 0.80 (95% confidence interval [0.65;0.98]), meaning that rectal cancer patients treated with the combination therapy benefit from a 20% risk reduction *on average*.

By relaxing the Weibull assumption (log-linear transformation *h*) to a Cox proportional hazards model (nonlinear transformation *h*), we obtain a hazard ratio of 0.78 (95% confidence interval [0.64–0.96]) and a marginal probabilistic index of 0.56 (95% confidence interval [0.51–0.61]), meaning that over all study centers, a randomly selected patient receiving Oxaliplatin has a 56% probability of staying disease-free longer than a randomly selected patient receiving the standard treatment only, given that they both have the same lymph node involvement and tumor grading.

## 4 Empirical evaluation

Practitioners interested in inference for marginal effects will likely apply some form of GEE estimation when analyzing a binary response, or might integrate over random effects in a conditional mixed-effects model for more complex response distributions. In this section, we assess the quality of likelihood-based marginal transformation inference (model mtram) in comparison to GEEs for binary responses and to mixed-effects models for continuous responses.

### 4.1 Data generating process

We simulate *N* = 100 clusters of five repeated measurements (*N_i_* = 5 and $U_{i}={\left(1,1,1,1,1\right)}^{⊤}$) from a logistic model (2.2) with $F=l\mathrm{ogi}t^{-1}$ and transformation function $h=\sqrt{1+\gamma_{1}^{2}}\cdot \text{logit}{}^{\circ}\chi_{9}^{2}$. The dependencies between repeated measurements in each cluster are described by $\Sigma_{i}={\left(\gamma_{1}^{2}\right)}_{5\times 5}+I_{5}$. We are interested in inference for the marginal effects $\mu :={\left(1+\gamma_{1}^{2}\right)}^{-1/2}\beta$ for various values of $\gamma_{1}\in \{0,0.5,1,1.5,2,3\}$. We simulated three uniform covariates ***X*** and defined $\beta ={\left(\beta_{1},\beta_{2},\beta_{3}\right)}^{⊤}={\left(0,1,2\right)}^{⊤}$. The baseline distribution (with $x={\left(0,0,0\right)}^{⊤}$) induces the same marginal $\chi_{9}^{2}$ laws for all five components with bivariate densities as depicted in Figure 1.

We report the mean-squared errors (MSEs) along with mean widths and coverages of 95% confidence intervals for $\mu_{p},p=1,2,3$ based on 10 000 simulation iterations in Table 3.

**Table 3:** Simulations. MSE, widths, and coverages of 95% confidence intervals for three marginal effects. For dichotomized binary responses, results obtained from GEEs can be directly compared to results from marginal transformation models (first two blocks). The last block reports results of marginal transformation models fitted to continuous responses

			$\gamma_{1}=0$	$\gamma_{1}=0.5$	$\gamma_{1}=1$	$\gamma_{1}=1.5$	$\gamma_{1}=2$	$\gamma_{1}=3$
GEE (exchangeable)	MSE	*μ*_1_	0.109	0.104	0.087	0.070	0.061	0.050
		*μ*_2_	0.111	0.106	0.091	0.076	0.067	0.062
		*μ*_3_	0.120	0.114	0.101	0.093	0.091	0.094
	CI width	*μ*_1_	1.273	1.247	1.141	1.035	0.958	0.868
		*μ*_2_	1.284	1.259	1.161	1.072	1.011	0.950
		*μ*_3_	1.317	1.295	1.219	1.169	1.153	1.165
	Coverage	*μ*_1_	0.948	0.947	0.947	0.950	0.948	0.947
		*μ*_2_	0.944	0.946	0.945	0.947	0.950	0.945
		*μ*_3_	0.942	0.944	0.947	0.945	0.942	0.941
mtram (binary)	MSE	*μ*_1_	0.109	0.104	0.086	0.068	0.057	0.044
		*μ*_2_	0.110	0.106	0.091	0.074	0.064	0.055
		*μ*_3_	0.119	0.114	0.100	0.091	0.087	0.088
	CI width	*μ*_1_	1.251	1.254	1.150	1.042	0.958	0.847
		*μ*_2_	1.276	1.268	1.172	1.079	1.014	0.942
		*μ*_3_	1.343	1.303	1.230	1.178	1.162	1.184
	Coverage	*μ*_1_	0.953	0.951	0.949	0.953	0.953	0.952
		*μ*_2_	0.948	0.950	0.949	0.951	0.954	0.953
		*μ*_3_	0.947	0.948	0.950	0.951	0.953	0.955
mtram (continuous)	MSE	*μ*_1_	0.074	0.067	0.045	0.029	0.019	0.009
		*μ*_2_	0.079	0.070	0.048	0.033	0.024	0.015
		*μ*_3_	0.082	0.075	0.056	0.046	0.038	0.033
	CI width	*μ*_1_	1.040	1.005	0.827	0.659	0.535	0.382
		*μ*_2_	1.061	1.020	0.853	0.705	0.602	0.485
		*μ*_3_	1.119	1.059	0.926	0.826	0.766	0.710
	Coverage	*μ*_1_	0.949	0.949	0.948	0.945	0.947	0.948
		*μ*_2_	0.945	0.948	0.949	0.948	0.947	0.951
		*μ*_3_	0.949	0.947	0.947	0.945	0.951	0.950

### 4.2 Binary responses

Binary responses were generated by dichotomization of the continuous response at the overall median. We fitted logistic GEEs with exchangeable working correlation structure and computed estimates and confidence intervals for all three marginal parameters $\mu_{p},p=1,2,3$. Results are shown in the first block of Table 3. In addition, marginal transformation models were fitted to these binary responses. Joint maximum-likelihood estimates of *γ*_1_ and β were computed from which we derived estimates and confidence intervals for the marginal effects $\mu_{p},p=1,2,3$. We drew 10 000 samples from the asymptotic joint normal distribution of *γ*_1_ and β to derive confidence intervals for $\mu_{p},p=1,2,3$ in each simulation iteration. These results in the second block of Table 3 are practically equivalent to the results reported for GEEs. For *μ*_2_ and *μ*_3_, the coverage of confidence intervals computed from model (2.2) were slightly closer to the nominal 95% level.

### 4.3 Continuous responses

Marginal transformation models fitted to data on the original scale, that is, without dichotomisation of the response, performed better in terms of smaller MSEs and confidence interval widths (third block in Table 3). The coverage remained close to the nominal level.

In addition, we compared mtrams for continuous responses to two mixed-effects models: a normal (LMM) and a conditional logistic mixed-effects transformation model (tramME, Tamási and Hothorn, 2021). Unlike GEEs, these two additional competitors are misspecified and one has to integrate over normal random effects to obtain a marginal distribution given a specific configuration of ***x***. For the normal LMM, the marginal distribution is again normal. Numerical integration was used to obtain marginal distributions from the tramME model.

**Fig. 4:**
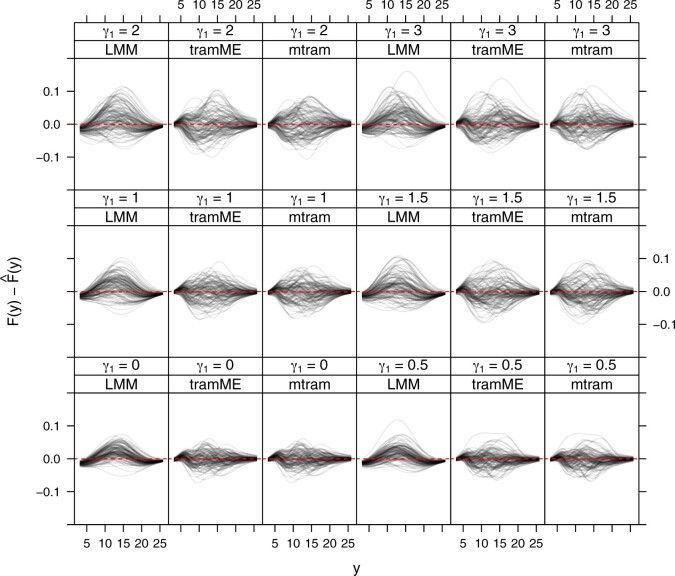
Difference between the true and estimated marginal distribution functions for a normal LMM (LMM), a conditional logistic mixed-effects transformation model (tramME) and a marginal transformation model (mtram). For mixed-effects models, the marginal distribution function was computed by integrating out the random effects (analytically for LMM and numerically for tramME).

For model (2.2), a conditional logistic mixed-effects transformation model with the same model complexity in terms of parameters for the transformation function and for the shift parameters, and a normal LMM, we derived the marginal distribution conditional on $x={\left(0.5,0.5,0.5\right)}^{⊤}$ for 100 simulation iterations and present the difference $F(y|x)-\hat{F}(y|x)$ of the true and estimated marginal distribution functions for all three procedures in Figure 4. The normal linear mixed-effects model (LMM) lead to biased marginal distributions, simply because the model is not able to adapt to the skewness of the marginal distributions. The results for the marginal (mtram) and conditional (tramME) transformation models were surprisingly similar, especially for smaller values of *γ*_1_. For $\gamma_{1}=0$ and thus independence measurements, results are expected to be identical. For $\gamma_{1}=3$, and thus very large correlations among the five repeated measurements, the estimated marginal distribution functions obtained from tramME seemed to be slightly more biased than the marginal distribution functions obtained from the mtram.

**Fig. 5:**
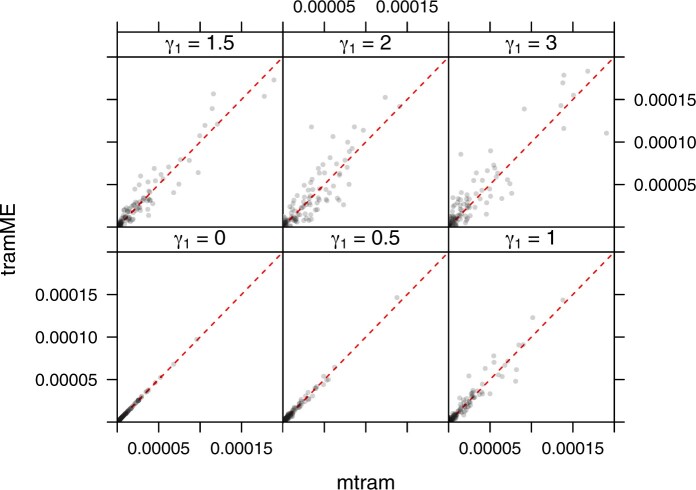
Integrated MSE between the true marginal distribution function and the estimated marginal distribution function for a conditional logistic mixed-effects transformation model (tramME) and a marginal logistic transformation model.

This impression is also supported in Figure 5, where the integrated MSE of the difference in distributions $\int_{-\infty }^{\infty }{\left(F(y|x)-\hat{F}(y|x)\right)}^{2}\cdot f(y|x)dy$ is presented for the conditional logistic mixed-effects transformation model (tramME) and the mtram (2.2). For $\gamma_{1}<2$, the two procedures performed very similar, for larger correlations the misspecified tramME model exhibited slightly larger discrepancies between true and estimated marginal distribution function. Of course, it is not possible to derive marginal effects and corresponding confidence intervals from such numerically obtained marginal distributions.

## 5 Discussion

There is a difference between a marginal and a marginally interpretable model. A marginal model, for example defined by generalized estimation equations (Zeger *and others*, 1988), does not specify the joint distribution. A marginally interpretable model is a model for the joint or conditional (given random effects) distribution from which one can infer the marginal distribution (Lee and Nelder, 2004). The models proposed here follow the latter approach with the important distinctive feature that very simple expressions for the marginal distribution function are available. Thus, there is no need to apply numerical integration to the joint or conditional model formulation. In our view, model (mtram) is especially attractive because it allows the interpretation of scaled regression coefficients as marginal effects acting on the marginal predictive distribution in terms of a log-odds ratio or a log-hazard ratio, for example. The Gaussian copula approach for obtaining marginally interpretable models has gained some interest in the last years (Zhang *and others*, 2021; Masarotto and Varin, 2012); however, the simple framework of transformation models allows estimation for a wide range of responses without encountering computational burdens or challenges that other methods typically do.

Naturally, the questions arises which model is to be preferred: a marginal, a conditional, or a marginally interpretable one? In this case, the “right” model is not the model which most closely reflects the data generating process, which is usually unknown, but rather the model that allows the user to answer the research question at hand by interpreting the estimated parameters, as McGee and Stringer (2022) point out. An advantage of transformation models is that besides allowing for interpretation of the fixed-effects on a marginal level, they also yield valid models for the whole marginal distribution (2.1) of the response given the covariates. An advantage of marginalized multilevel models (Heagerty and Zeger, 2000) over marginal transformation models is that the former models are parameterized in terms of marginal effects of interest, whereas effect shrinkage is part of the latter models. The distribution-free nature, general applicability to all types of responses, and the relative computational simplicity are, in our opinion, attractive features of transformation models compared to marginalized multilevel models.

The models and estimation procedures introduced here are limited by some practical and some conceptual constraints. Response-varying regression coefficients β(y) define distribution regression models (Foresi and Peracchi, 1995; Chernozhukov *and others*, 2013), where corresponding mixed-effects models have been presented recently (Garcia *and others*, 2019). This would be relatively straightforward to implement in the framework presented here, in fact, stratification in Weibull models was parameterized in a similar way. A mix of continuous and censored observations within one cluster would require to compute the likelihood by partial integration over an *N_i_*-dimensional normal, this is currently not implemented. On a more conceptual level, it seems impossible to implement multilevel models for discrete or censored responses, because the likelihood (2.7) is only defined for contributions by independent clusters.

## 6 Computational details

The empirical analyses presented in Sections 3 and 4 are reproducible using the mtram package vignette (Online Appendix, Barbanti and Hothorn, 2022) in package **tram** (Hothorn *and others*, 2022). Infrastructure for transformation models from package **mlt** was used to define marginal models. Augmented Lagrangian Minimization implemented in the auglag() function of package **alabama** (Varadhan, 2022) was used for optimizing the log-likelihood. Numerical integration to compute the discrete and censored version of the log-likelihood was performed by **SparseGrid** (Ypma, 2013). GEEs were estimated using package **geepack** (Højsgaard *and others*, 2022) and conditional mixed-effects (LMM and tramME) models using package **tramME** (Tamasi, 2022). Packages **lme4** (Bates *and others*, 2015) and **glmmTMB** (Brooks *and others*, 2017) were used to fit generalized mixed-effects models. All results were obtained using R version 4.2.2 (R Core Team, 2022).

## Appendix

### A Likelihood function: censored and discrete case

The *i*th contribution to the likelihood (2.6) is given by the *N_i_*-dimensional normal integral

$$\text{exp}(\ell_{i}(\vartheta ,\beta ,\gamma ))=\int_{z({\underline{y}}_{i}|\vartheta ,\beta ,\gamma )}^{z({\bar{y}}_{i}|\vartheta ,\beta ,\gamma )}\phi_{N_{i}}\left(z,0_{N_{i}},U_{i}\Lambda (\gamma )\Lambda {\left(\gamma \right)}^{⊤}U_{i}^{⊤}+I_{N_{i}}\right)dz.$$

With $D_{i}(\gamma )=\text{diag}(U_{i}\Lambda (\gamma )\Lambda {\left(\gamma \right)}^{⊤}U_{i}^{⊤}+I_{N_{i}})\cdot I_{N_{i}}$ we obtain the corresponding correlation matrix as

$$\begin{array}{c} C_{i}(\gamma )=D_{i}{\left(\gamma \right)}^{-1/2}\Sigma_{i}(\gamma )D_{i}{\left(\gamma \right)}^{-1/2}=V_{i}(\gamma )V_{i}{\left(\gamma \right)}^{⊤}+D_{i}{\left(\gamma \right)}^{-1}\in R^{N_{i}\times N_{i}}\\ V_{i}(\gamma )=D_{i}{\left(\gamma \right)}^{-1/2}U_{i}\Lambda (\gamma )\in R^{N_{i}\times R} \end{array}$$

and the integration limits become (again with z() defined in (2.5))

$$\underline{z}=D_{i}{\left(\gamma \right)}^{-1/2}z({\underline{y}}_{i}|\vartheta ,\beta ,\gamma )\;\text{and}\;\bar{z}=D_{i}{\left(\gamma \right)}^{-1/2}z({\bar{y}}_{i}|\vartheta ,\beta ,\gamma ).$$

According to Marsaglia (1963), the above normal probability can be written as

$$\int_{\underline{z}}^{\bar{z}}\phi_{N_{i}}\left(z,0_{N_{i}},C_{i}(\gamma )\right)dz=\int_{R^{R}}\phi_{R}(w,0_{R},I_{R})\int_{\underline{z}-\mathrm{Vw}}^{\bar{z}-\mathrm{Vw}}\phi_{N_{i}}\left(y,0_{N_{i}},D_{i}{\left(\gamma \right)}^{-1}\right)dwdy$$

and can, following Genz and Bretz (2009) here, further be simplified to

$$\begin{array}{c} =\int_{R^{R}}\phi_{R}(w,0_{R},I_{R})\prod_{ı=1}^{N_{i}}\left[\Phi \left(\frac{{\bar{z}}_{ı}-\sum_{r=1}^{R}v_{ır}w_{r}}{\sqrt{d_{i}}}\right)-\Phi \left(\frac{{\underline{z}}_{ı}-\sum_{r=1}^{R}v_{ır}w_{r}}{\sqrt{d_{i}}}\right)\right]dw\\ \overset{w=\Phi_{R}^{-1}(q)}{=}\int_{{\left[0,1\right]}^{R}}\prod_{ı=1}^{N_{i}}\left[\Phi \left(\frac{{\bar{z}}_{ı}-\sum_{r=1}^{R}v_{ır}\Phi^{-1}(q_{r})}{\sqrt{d_{ı}}}\right)-\Phi \left(\frac{{\underline{z}}_{ı}-\sum_{r=1}^{R}v_{ır}\Phi^{-1}(q_{r})}{\sqrt{d_{ı}}}\right)\right]dq. \end{array}$$

The elements $d_{ı}$ are the diagonal elements of $D_{i}{\left(\gamma \right)}^{-1}$ and thus standardization of *z* and $U_{i}\Lambda (\gamma )$ cancel out in this case such that we get

$$=\int_{{\left[0,1\right]}^{R}}\prod_{ı=1}^{N_{i}}\left[\Phi \left({\tilde{\bar{z}}}_{ı}-\sum_{r=1}^{R}{\tilde{v}}_{ır}\Phi^{-1}(q_{r})\right)-\Phi \left({\tilde{\underline{z}}}_{ı}-\sum_{r=1}^{R}{\tilde{v}}_{ır}\Phi^{-1}(q_{r})\right)\right]dq$$

with ${\tilde{\bar{z}}}_{ı}$ and ${\tilde{\underline{z}}}_{ı}$ being the elements of $z({\bar{y}}_{i}|\vartheta ,\beta ,\gamma )$ and $z({\underline{y}}_{i}|\vartheta ,\beta ,\gamma )$, respectively, and ${\tilde{v}}_{ır}$ are the elements of $U_{i}\Lambda (\gamma )$.

The latter expression is an *R*-dimensional integral over the unit cube (random intercept models have *R* = 1 and correlated random intercept/random slope models correspond to *R* = 3) of products of univariate normal probabilities. It should be noted that, unlike using an Laplace or other approximation of the likelihood, the above term is the exact likelihood contribution. It can be approximated up to any desired accuracy using numerical integration procedures. An analytic expression for the score function seems quite challenging and one thus has to rely on numerical approaches such as sparse grids (Heiss and Winschel, 2008).

### B Likelihood and score function: continuous case

The joint probability of $y_{i}\in R^{N_{i}}$ is given by:

$$P(Y_{i}\leq y_{i}|X_{i},U_{i})=\Phi_{0_{N_{i}},\Sigma_{i}(\gamma )}\left(D_{i}(\gamma )\Phi_{N_{i}}^{-1}\left(F_{N_{i}}\{D_{i}{\left(\gamma \right)}^{-1}[A(y_{i})\vartheta -X_{i}\beta ]\}\right)\right).$$

To simplify the notation, we define z() as in (2.5):

$$z(y_{i}|\vartheta ,\beta ,\gamma )=D_{i}(\gamma )\Phi_{N_{i}}^{-1}(F_{N_{i}}\{D_{i}{\left(\gamma \right)}^{-1}[A(y_{i})\vartheta -X_{i}\beta ]\}).$$

We can derive the corresponding joint density for an arbitrary *F*:

$$\begin{array}{c} f_{Y_{i}}(y_{i}|\vartheta ,\beta ,\gamma )={\left({\left(2\pi \right)}^{N_{i}}\left|L_{i}(\gamma )L_{i}{\left(\gamma \right)}^{⊤}\right|\right)}^{-1/2}\times \\ \;\;\text{exp}\;\left(-\frac{1}{2}{\left\|z{\left(y_{i}|\vartheta ,\beta ,\gamma \right)}^{⊤}L_{i}{\left(\gamma \right)}^{-1}\right\|}_{2}^{2}\right)\times \\ \;\prod_{ı=1}^{N_{i}}\frac{D_{i}{\left(\gamma \right)}_{ıı}f\{{\left(D_{i}{\left(\gamma \right)}^{-1}\right)}_{ıı}[a{\left(y_{iı}\right)}^{⊤}\vartheta -X_{i}\beta ]\}}{\phi (\Phi^{-1}\left(F\{D_{i}{\left(\gamma \right)}^{-1}[a{\left(y_{iı}\right)}^{⊤}\vartheta -X_{i}\beta ]\}\right))}{\left(D_{i}{\left(\gamma \right)}^{-1}\right)}_{ıı}a\prime {\left(y_{iı}\right)}^{⊤}\vartheta \\ ={\left|L_{i}(\gamma )L_{i}{\left(\gamma \right)}^{⊤}\right|}^{-1/2}\times \\ \;\;\text{exp}\;\left(-\frac{1}{2}{\left\|z{\left(y_{i}|\vartheta ,\beta ,\gamma \right)}^{⊤}L_{i}{\left(\gamma \right)}^{-1}\right\|}_{2}^{2}\right)\times \\ \;\;\text{exp}\;\left(\frac{1}{2}{\left\|D_{i}{\left(\gamma \right)}^{-1}z(y_{i}|\vartheta ,\beta ,\gamma )\right\|}_{2}^{2}\right)\times \\ \;\prod_{ı=1}^{N_{i}}f\{{\left(D_{i}{\left(\gamma \right)}^{-1}\right)}_{ıı}[a{\left(y_{iı}\right)}^{⊤}\vartheta -X_{i}\beta ]\}a\prime {\left(y_{iı}\right)}^{⊤}\vartheta \\ ={\left|L_{i}(\gamma )L_{i}{\left(\gamma \right)}^{⊤}\right|}^{-1/2}\times \\ \;\;\text{exp}\;\left(-\frac{1}{2}z{\left(y_{i}|\vartheta ,\beta ,\gamma \right)}^{⊤}\Sigma_{i}{\left(\gamma \right)}^{-1}z(y_{i}|\vartheta ,\beta ,\gamma )\right)\times \\ \;\;\text{exp}\;\left(\frac{1}{2}z{\left(y_{i}|\vartheta ,\beta ,\gamma \right)}^{⊤}D_{i}{\left(\gamma \right)}^{-2}z(y_{i}|\vartheta ,\beta ,\gamma )\right)\times \\ \;\prod_{ı=1}^{N_{i}}f\{{\left(D_{i}{\left(\gamma \right)}^{-1}\right)}_{ıı}[a{\left(y_{iı}\right)}^{⊤}\vartheta -X_{i}\beta ]\}a\prime {\left(y_{iı}\right)}^{⊤}\vartheta . \end{array}$$

The resulting log-likelihood contribution (2.7) for the *i*th observation is given by:

$$\begin{array}{c} \ell_{i}(\vartheta ,\beta ,\gamma )\approx \;\text{log}(f_{Y_{i}}(y_{i}|\vartheta ,\beta ,\gamma ))\\ =-\frac{1}{2}\text{log}\;\left|L_{i}(\gamma )L_{i}{\left(\gamma \right)}^{⊤}\right|-\frac{1}{2}{\left\|z{\left(y_{i}|\vartheta ,\beta ,\gamma \right)}^{⊤}L_{i}{\left(\gamma \right)}^{-1}\right\|}_{2}^{2}+\\ \;\frac{1}{2}{\left\|D_{i}{\left(\gamma \right)}^{-1}z{\left(y_{i}|\vartheta ,\beta ,\gamma \right)}^{⊤}\right\|}_{2}^{2}+\\ \;\;{\text{log}}_{N_{i}}{\left(f_{N_{i}}\{D_{i}{\left(\gamma \right)}^{-1}[A(y_{i})\vartheta -X_{i}\beta ]\}\right)}^{⊤}1_{N_{i}}+\\ \;\;{\text{log}}_{N_{i}}{\left(A\prime (y_{i})\vartheta \right)}^{⊤}1_{N_{i}}\\ =-\frac{1}{2}\text{log}\;\left|\Sigma_{i}(\gamma )\right|+\\ -\frac{1}{2}z{\left(y_{i}|\vartheta ,\beta ,\gamma \right)}^{⊤}(\Sigma_{i}{\left(\gamma \right)}^{-1}-D_{i}{\left(\gamma \right)}^{-2})z(y_{i}|\vartheta ,\beta ,\gamma )+\\ \;\;{\text{log}}_{N_{i}}{\left(f_{N_{i}}\{D_{i}{\left(\gamma \right)}^{-1}[A(y_{i})\vartheta -X_{i}\beta ]\}\right)}^{⊤}1_{N_{i}}+\\ \;\;{\text{log}}_{N_{i}}{\left(A\prime (y_{i})\vartheta \right)}^{⊤}1_{N_{i}}. \end{array}$$

The score function for all model parameters ϑ,β, and γ can be derived based on the results of Stroup (2012) as applied to normal linear mixed-effects models by Wang and Merkle (2018).

With the M=R(R+1)/2 unique elements $\gamma ={\left(\gamma_{1},\ldots ,\gamma_{M}\right)}^{⊤}$ of the lower Cholesky factor Λ(γ) we get

$$\frac{\partial \Sigma_{i}(\gamma )}{\partial \gamma_{m}}=U_{i}\frac{\partial \Lambda (\gamma )\Lambda {\left(\gamma \right)}^{⊤}}{\partial \gamma_{m}}U_{i}^{⊤}=U_{i}\left(\frac{\partial \Lambda (\gamma )}{\partial \gamma_{m}}\Lambda {\left(\gamma \right)}^{⊤}+\Lambda (\gamma )\frac{\partial \Lambda {\left(\gamma \right)}^{⊤}}{\partial \gamma_{m}}\right)U_{i}^{⊤}.$$

The derivative of Λ(γ) with respect to an element *γ_m_* of γ is a matrix of zeros with the exception of a single one at the position of *γ_m_*. Moreover, we compute:

$$\begin{array}{c} \frac{\partial D_{i}(\gamma )}{\partial \gamma_{m}}=\frac{1}{2}D_{i}{\left(\gamma \right)}^{-1}\text{diag}\left(U_{i}\left(\frac{\partial \Lambda (\gamma )}{\partial \gamma_{m}}\Lambda {\left(\gamma \right)}^{⊤}+\Lambda (\gamma )\frac{\partial \Lambda {\left(\gamma \right)}^{⊤}}{\partial \gamma_{m}}\right)U_{i}^{⊤}\right)\cdot I_{N_{i}}\\ =\frac{1}{2}D_{i}{\left(\gamma \right)}^{-1}\text{diag}\left(\frac{\partial \Sigma_{i}(\gamma )}{\partial \gamma_{m}}\right)\cdot I_{N_{i}}\\ \frac{\partial D_{i}{\left(\gamma \right)}^{-1}}{\partial \gamma_{m}}=-\frac{1}{2}{\left(D_{i}{\left(\gamma \right)}^{-1}\right)}^{3}\text{diag}\left(\frac{\partial \Sigma_{i}(\gamma )}{\partial \gamma_{m}}\right)\cdot I_{N_{i}}\\ \frac{\partial {\left(D_{i}{\left(\gamma \right)}^{-1}\right)}^{2}}{\partial \gamma_{m}}=-{\left(D_{i}{\left(\gamma \right)}^{-1}\right)}^{4}\text{diag}\left(\frac{\partial \Sigma_{i}(\gamma )}{\partial \gamma_{m}}\right)\cdot I_{N_{i}}\\ =-{\left(\text{diag}(\Sigma_{i}(\gamma ))\right)}^{-2}\text{diag}\left(\frac{\partial \Sigma_{i}(\gamma )}{\partial \gamma_{m}}\right)\cdot I_{N_{i}} \end{array}$$

and

$$\begin{array}{c} \frac{\partial z(y_{i}|\vartheta ,\beta ,\gamma )}{\partial \gamma_{m}}=\frac{\partial D_{i}(\gamma )\Phi_{N_{i}}^{-1}(F_{N_{i}}\{D_{i}{\left(\gamma \right)}^{-1}[A(y_{i})\vartheta -X_{i}\beta ]\})}{\partial \gamma_{m}}\\ =\left(\frac{\partial }{\partial \gamma_{m}}D_{i}(\gamma )\right)\Phi_{N_{i}}^{-1}(F_{N_{i}}\{D_{i}{\left(\gamma \right)}^{-1}[A(y_{i})\vartheta -X_{i}\beta ]\})+\\ \;\;+D_{i}(\gamma )\left(\frac{\partial }{\partial \gamma_{m}}\Phi_{N_{i}}^{-1}(F_{N_{i}}\{D_{i}{\left(\gamma \right)}^{-1}[A(y_{i})\vartheta -X_{i}\beta ]\})\right)\\ =\frac{1}{2}D_{i}{\left(\gamma \right)}^{-1}\text{diag}\left(\frac{\partial \Sigma_{i}(\gamma )}{\partial \gamma_{m}}\right)\cdot I_{N_{i}}\Phi_{N_{i}}^{-1}(F_{N_{i}}\{D_{i}{\left(\gamma \right)}^{-1}[A(y_{i})\vartheta -X_{i}\beta ]\})+\\ \;\;+D_{i}(\gamma )\frac{f_{N_{i}}\{D_{i}{\left(\gamma \right)}^{-1}[A(y_{i})\vartheta -X_{i}\beta ]\}}{\phi_{N_{i}}[\Phi_{N_{i}}^{-1}(F_{N_{i}}\{D_{i}{\left(\gamma \right)}^{-1}[A(y_{i})\vartheta -X_{i}\beta ]\})]}[A(y_{i})\vartheta -X_{i}\beta ]\frac{\partial D_{i}{\left(\gamma \right)}^{-1}}{\partial \gamma_{m}}\\ =\frac{1}{2}D_{i}{\left(\gamma \right)}^{-2}\text{diag}\left(\frac{\partial \Sigma_{i}(\gamma )}{\partial \gamma_{m}}\right)\cdot I_{N_{i}}z(y_{i}|\vartheta ,\beta ,\gamma )+\\ \;\;-\frac{1}{2}D_{i}{\left(\gamma \right)}^{-2}\text{diag}\left(\frac{\partial \Sigma_{i}(\gamma )}{\partial \gamma_{m}}\right)\cdot I_{N_{i}}\frac{f_{N_{i}}\{D_{i}{\left(\gamma \right)}^{-1}[A(y_{i})\vartheta -X_{i}\beta ]\}[A(y_{i})\vartheta -X_{i}\beta ]}{\phi_{N_{i}}[\Phi_{N_{i}}^{-1}(F_{N_{i}}\{D_{i}{\left(\gamma \right)}^{-1}[A(y_{i})\vartheta -X_{i}\beta ]\})]}\\ =\frac{1}{2}D_{i}{\left(\gamma \right)}^{-2}\text{diag}\left(\frac{\partial \Sigma_{i}(\gamma )}{\partial \gamma_{m}}\right)\cdot I_{N_{i}}\times \\ \;\;\left[z(y_{i}|\vartheta ,\beta ,\gamma )-\frac{f_{N_{i}}\{D_{i}{\left(\gamma \right)}^{-1}[A(y_{i})\vartheta -X_{i}\beta ]\}[A(y_{i})\vartheta -X_{i}\beta ]}{\phi_{N_{i}}[\Phi_{N_{i}}^{-1}(F_{N_{i}}\{D_{i}{\left(\gamma \right)}^{-1}[A(y_{i})\vartheta -X_{i}\beta ]\})]}\right] \end{array}$$

Thus,

$$\begin{array}{c} \frac{\partial \ell_{i}(\vartheta ,\beta ,\gamma )}{\partial \gamma_{m}}=-\frac{1}{2}tr\left(\Sigma_{i}{\left(\gamma \right)}^{-1}\frac{\partial \Sigma_{i}(\gamma )}{\partial \gamma_{m}}\right)+\\ -\frac{1}{2}[\left(\frac{\partial z{\left(y_{i}|\vartheta ,\beta ,\gamma \right)}^{⊤}}{\partial \gamma_{m}}\right)(\Sigma_{i}{\left(\gamma \right)}^{-1}-D_{i}{\left(\gamma \right)}^{-2})z(y_{i}|\vartheta ,\beta ,\gamma )+\\ \;\;\;\;z{\left(y_{i}|\vartheta ,\beta ,\gamma \right)}^{⊤}\left(\frac{\partial }{\partial \gamma_{m}}(\Sigma_{i}{\left(\gamma \right)}^{-1}-D_{i}{\left(\gamma \right)}^{-2})\right)z(y_{i}|\vartheta ,\beta ,\gamma )+\\ \;\;\;\;z{\left(y_{i}|\vartheta ,\beta ,\gamma \right)}^{⊤}(\Sigma_{i}{\left(\gamma \right)}^{-1}-D_{i}{\left(\gamma \right)}^{-2})\left(\frac{\partial }{\partial \gamma_{m}}z(y_{i}|\vartheta ,\beta ,\gamma )\right)]+\\ +\frac{f_{N_{i}}\prime (D_{i}{\left(\gamma \right)}^{-1}[A(y_{i})\vartheta -X_{i}\beta ])}{f_{N_{i}}(D_{i}{\left(\gamma \right)}^{-1}[A(y_{i})\vartheta -X_{i}\beta ])}[A(y_{i})\vartheta -X_{i}\beta ]\frac{\partial D_{i}{\left(\gamma \right)}^{-1}}{\partial \gamma_{m}}\\ =-\frac{1}{2}tr\left(\Sigma_{i}{\left(\gamma \right)}^{-1}\frac{\partial \Sigma_{i}(\gamma )}{\partial \gamma_{m}}\right)+\\ -\frac{1}{2}[\left(\frac{\partial z{\left(y_{i}|\vartheta ,\beta ,\gamma \right)}^{⊤}}{\partial \gamma_{m}}\right)(\Sigma_{i}{\left(\gamma \right)}^{-1}-D_{i}{\left(\gamma \right)}^{-2})z(y_{i}|\vartheta ,\beta ,\gamma )+\\ \;\;\;\;-z{\left(y_{i}|\vartheta ,\beta ,\gamma \right)}^{⊤}(\Sigma_{i}{\left(\gamma \right)}^{-1}\frac{\partial \Sigma_{i}(\gamma )}{\partial \gamma_{m}}\Sigma_{i}{\left(\gamma \right)}^{-1}+\\ \;\;{\left(D_{i}{\left(\gamma \right)}^{-1}\right)}^{4}\text{diag}\left(\frac{\partial \Sigma_{i}(\gamma )}{\partial \gamma_{m}}\right)\cdot I_{N_{i}})z(y_{i}|\vartheta ,\beta ,\gamma )+\\ \;\;\;\;z{\left(y_{i}|\vartheta ,\beta ,\gamma \right)}^{⊤}(\Sigma_{i}{\left(\gamma \right)}^{-1}-D_{i}{\left(\gamma \right)}^{-2})\left(\frac{\partial z(y_{i}|\vartheta ,\beta ,\gamma )}{\partial \gamma_{m}}\right)]+\\ \;+\frac{f_{N_{i}}\prime (D_{i}{\left(\gamma \right)}^{-1}[A(y_{i})\vartheta -X_{i}\beta ])}{f_{N_{i}}(D_{i}{\left(\gamma \right)}^{-1}[A(y_{i})\vartheta -X_{i}\beta ])}[A(y_{i})\vartheta -X_{i}\beta ]\frac{\partial D_{i}{\left(\gamma \right)}^{-1}}{\partial \gamma_{m}}\\ \frac{\partial \ell_{i}(\vartheta ,\beta ,\gamma )}{\partial \beta }=z{\left(y_{i}|\vartheta ,\beta ,\gamma \right)}^{⊤}(\Sigma_{i}{\left(\gamma \right)}^{-1}-D_{i}{\left(\gamma \right)}^{-2})\times \\ \;\frac{f_{N_{i}}(D_{i}{\left(\gamma \right)}^{-1}[A(y_{i})\vartheta -X_{i}\beta ])}{\phi_{N_{i}}(\Phi_{N_{i}}^{-1}(F_{N_{i}}\{D_{i}{\left(\gamma \right)}^{-1}[A(y_{i})\vartheta -X_{i}\beta ]\}))}X_{i}+\\ \;-1_{N_{i}}^{⊤}\left(\frac{f_{N_{i}}^{\prime }(D_{i}{\left(\gamma \right)}^{-1}[A(y_{i})\vartheta -X_{i}\beta ])}{f_{N_{i}}(D_{i}{\left(\gamma \right)}^{-1}[A(y_{i})\vartheta -X_{i}\beta ])}D_{i}{\left(\gamma \right)}^{-1}X_{i}\right)\\ \frac{\partial \ell_{i}(\vartheta ,\beta ,\gamma )}{\partial \vartheta }=-z{\left(y_{i}|\vartheta ,\beta ,\gamma \right)}^{⊤}(\Sigma_{i}{\left(\gamma \right)}^{-1}-D_{i}{\left(\gamma \right)}^{-2})\times \\ \;\frac{f_{N_{i}}(D_{i}{\left(\gamma \right)}^{-1}[A(y_{i})\vartheta -X_{i}\beta ])}{\phi_{N_{i}}(\Phi_{N_{i}}^{-1}(F_{N_{i}}\{D_{i}{\left(\gamma \right)}^{-1}[A(y_{i})\vartheta -X_{i}\beta ]\}))}A(y_{i})+\\ \;1_{N_{i}}^{⊤}\left(\frac{f_{N_{i}}^{\prime }(D_{i}{\left(\gamma \right)}^{-1}[A(y_{i})\vartheta -X_{i}\beta ])}{f_{N_{i}}(D_{i}{\left(\gamma \right)}^{-1}[A(y_{i})\vartheta -X_{i}\beta ])}D_{i}{\left(\gamma \right)}^{-1}A(y_{i})\right)+1_{N_{i}}^{⊤}\frac{1}{A\prime (y_{i})\vartheta }A\prime (y_{i}). \end{array}$$

For $F=\Phi ,\;z(y_{i}|\vartheta ,\beta ,\gamma )=A(y_{i})\vartheta -X_{i}\beta$, the joint distribution simplifies to

$$P(Y_{i}\leq y_{i}|X_{i},U_{i})=\Phi_{0_{N_{i}},\Sigma_{i}(\gamma )}\left(A(y_{i})\vartheta -X_{i}\beta \right)$$

and the joint density becomes:

$$\begin{array}{c} f_{Y_{i}}(y_{i}|\vartheta ,\beta ,\gamma )={\left({\left(2\pi \right)}^{N_{i}}\left|L_{i}(\gamma )L_{i}{\left(\gamma \right)}^{⊤}\right|\right)}^{-1/2}\times \\ \;\;\text{exp}\;\left(-\frac{1}{2}{\left(A(y_{i})\vartheta -X_{i}\beta \right)}^{⊤}\Sigma_{i}{\left(\gamma \right)}^{-1}(A(y_{i})\vartheta -X_{i}\beta )\right)\times \\ \;\prod_{ı=1}^{N_{i}}a\prime {\left(y_{iı}\right)}^{⊤}\vartheta . \end{array}$$

We obtain the corresponding log-likelihood:

$$\begin{array}{c} \ell_{i}(\vartheta ,\beta ,\gamma )\approx \;\text{log}(f_{Y_{i}}(y_{i}|\vartheta ,\beta ,\gamma ))\\ \propto -\frac{1}{2}\text{log}\;\left|\Sigma_{i}(\gamma )\right|+\\ \;-\frac{1}{2}{\left(A(y_{i})\vartheta -X_{i}\beta \right)}^{⊤}\Sigma_{i}{\left(\gamma \right)}^{-1}(A(y_{i})\vartheta -X_{i}\beta )+\\ \;\;{\text{log}}_{N_{i}}{\left(A\prime (y_{i})\vartheta \right)}^{⊤}1_{N_{i}}\\ =-\frac{1}{2}\text{log}\;\left|\Sigma_{i}(\gamma )\right|-\frac{1}{2}\vartheta^{⊤}A{\left(y_{i}\right)}^{⊤}\Sigma_{i}{\left(\gamma \right)}^{-1}A(y_{i})\vartheta +\vartheta^{⊤}A{\left(y_{i}\right)}^{⊤}\Sigma_{i}{\left(\gamma \right)}^{-1}X_{i}\beta +\\ -\frac{1}{2}\beta^{⊤}X_{i}^{⊤}\Sigma_{i}{\left(\gamma \right)}^{-1}X_{i}\beta +{\text{log}}_{N_{i}}{\left(A\prime (y_{i})\vartheta \right)}^{⊤}1_{N_{i}}. \end{array}$$

The scores are:

$$\begin{array}{c} \frac{\partial \ell_{i}(\vartheta ,\beta ,\gamma )}{\partial \gamma_{m}}=-\frac{1}{2}tr\left(\Sigma_{i}{\left(\gamma \right)}^{-1}\frac{\partial \Sigma_{i}(\gamma )}{\partial \gamma_{m}}\right)+\\ \frac{1}{2}{\left(A(y_{i})\vartheta -X_{i}\beta \right)}^{⊤}\Sigma_{i}{\left(\gamma \right)}^{-1}\frac{\partial \Sigma_{i}(\gamma )}{\partial \gamma_{m}}\Sigma_{i}{\left(\gamma \right)}^{-1}(A(y_{i})\vartheta -X_{i}\beta )\\ \frac{\partial \ell_{i}(\vartheta ,\beta ,\gamma )}{\partial \beta }=\vartheta^{⊤}A{\left(y_{i}\right)}^{⊤}\Sigma_{i}{\left(\gamma \right)}^{-1}X_{i}-\beta^{⊤}X_{i}^{⊤}\Sigma_{i}{\left(\gamma \right)}^{-1}X_{i}\\ \frac{\partial \ell_{i}(\vartheta ,\beta ,\gamma )}{\partial \vartheta }=-\vartheta^{⊤}A{\left(y_{i}\right)}^{⊤}\Sigma_{i}{\left(\gamma \right)}^{-1}A(y_{i})+\beta^{⊤}X_{i}^{⊤}\Sigma_{i}{\left(\gamma \right)}^{-1}A(y_{i})+1_{N_{i}}^{⊤}\frac{1}{A\prime (y_{i})\vartheta }A\prime (y_{i}). \end{array}$$

## References

[kxac048-B1] Aalen O. O. , Borgan Ø., Gjessing H. K. (2008). Survival and Event History Analysis . New York, USA: Springer.

[kxac048-B2] Backer M. D. , Vroey C. D., Lesaffre E., Scheys I., Keyser P. D. (1998). Twelve weeks of continuous oral therapy for toenail onychomycosis caused by dermatophytes: a double-blind comparative trial of terbinafine 250 mg/day versus itraconazole 200 mg/day. Journal of the American Academy of Dermatology 38, S57–S63. 9594939 10.1016/s0190-9622(98)70486-4

[kxac048-B3] Barbanti L. , Hothorn T. (2022). *Some Applications of Marginally Interpretable Linear Transformation Models for Clustered Observations .* R package vignette version 0.8-0. https://CRAN.R-project.org/package=tram

[kxac048-B4] Bates D. , Mächler M., Bolker B., Walker S. (2015). Fitting linear mixed-effects models using **lme4**. Journal of Statistical Software 67, 1–48.

[kxac048-B5] Belenky G. , Wesensten N. J., Thorne D. R., Thomas M. L., Sing H. C., Redmond D. P., Russo M. B., Balkin T. J. (2003). Patterns of performance degradation and restoration during sleep restriction and subsequent recovery: a sleep dose-response study. Journal of Sleep Research 12, 1–12. 12603781 10.1046/j.1365-2869.2003.00337.x

[kxac048-B6] Brooks M. E. , Kristensen K., van Benthem K. J., Magnusson A., Berg C. W., Nielsen A., Skaug H. J., Mächler M., Bolker B. M. (2017). glmmTMB balances speed and flexibility among packages for zero-inflated generalized linear mixed modeling. The R Journal 9, 378–400.

[kxac048-B7] Cai T. , Wei L. J., Wilcox M. (2000). Semiparametric regression analysis for clustered failure time data. Biometrika 87, 867–878.

[kxac048-B8] Chernozhukov V. , Fernández-Val I., Melly B. (2013). Inference on counterfactual distributions. Econometrica 81, 2205–2268.

[kxac048-B9] Chow R. T. , Heller G. Z., Barnsley L. (2006). The effect of 300 mW, 830 nm laser on chronic neck pain: a double-blind, randomized, placebo-controlled study. Pain 124, 201–210. 16806710 10.1016/j.pain.2006.05.018

[kxac048-B10] Foresi S. , Peracchi F. (1995). The conditional distribution of excess returns: an empirical analysis. Journal of the American Statistical Association 90, 451–466.

[kxac048-B11] Garcia T. P. , Marder K., Wang Y. (2019). Time-varying proportional odds model for mega-analysis of clustered event times. Biostatistics 20, 129–146. 29309509 10.1093/biostatistics/kxx065 PMC6402758

[kxac048-B12] Genz A. , Bretz F. (2009). Computation of Multivariate Normal and t Probabilities, Lecture Notes in Statistics. Heidelberg: Springer.

[kxac048-B13] Goethals K. , Janssen P., Duchateau L. (2008). Frailty models and copulas: similarities and differences. Journal of Applied Statistics 35, 1071–1079.

[kxac048-B14] Gory J. J. , Craigmile P. F., MacEachern S. N. (2021). A class of generalized linear mixed models adjusted for marginal interpretability. Statistics in Medicine 40, 427–440. 33094523 10.1002/sim.8782

[kxac048-B15] Gurka M. J. , Edwards L. J., Muller K. E., Kupper L. L. (2006). Extending the Box-Cox transformation to the linear mixed model. Journal of the Royal Statistical Society: Series A (Statistics in Society*)* 169, 273–288.

[kxac048-B16] Heagerty P. J . (1999). Marginally specified logistic-normal models for longitudinal binary data. Biometrics 55, 688–698. 11314994 10.1111/j.0006-341x.1999.00688.x

[kxac048-B17] Heagerty P. J. , Zeger S. L. (2000). Marginalized multilevel models and likelihood inference (with comments and a rejoinder by the authors). Statistical Science 15, 1–26.

[kxac048-B18] Heiss F. , Winschel V. (2008). Likelihood approximation by numerical integration on sparse grids. Journal of Econometrics 144, 62–80.

[kxac048-B19] Hothorn T. (2020). Most likely transformations: the mlt package. Journal of Statistical Software 92, 1–68.

[kxac048-B20] Hothorn T. , Barbanti L., Siegfried S. (2022). *tram: Transformation Models .* R package version 0.8-0. https://CRAN.R-project.org/package=tram

[kxac048-B21] Hothorn T. , Kneib T., Bühlmann P. (2014). Conditional transformation models. Journal of the Royal Statistical Society: Series B (Statistical Methodology) 76, 3–27.

[kxac048-B22] Hothorn T. , Möst L., Bühlmann P. (2018). Most likely transformations. Scandinavian Journal of Statistics 45, 110–134.

[kxac048-B23] Hutmacher M. M. , French J. L., Krishnaswami S., Menon S. (2011). Estimating transformations for repeated measures modeling of continuous bounded outcome data. Statistics in Medicine 30, 935–949. 21472758 10.1002/sim.4155

[kxac048-B24] Højsgaard S. , Halekoh U., Yan J. (2022). *geepack: Generalized Estimating Equation Package .* R package version 1.3.9. https://CRANR-project.org/package=geepack

[kxac048-B25] Klein N. , Hothorn T., Barbanti L., Kneib T. (2022). Multivariate conditional transformation models. Scandinavian Journal of Statistics 49, 116–142.

[kxac048-B26] Lee Y. , Nelder J. A. (2004). Conditional and marginal models: another view. Statistical Science 19, 219–238.

[kxac048-B27] Lin Y. , Luo Y., Xie S., Chen K. (2017). Robust rank estimation for transformation models with random effects. Biometrika 104, 971–986.

[kxac048-B28] Lindsey J. K. (1999). Some statistical heresies. Journal of the Royal Statistical Society: Series D (The Statistician) 48, 1–40.

[kxac048-B29] Manuguerra M. , Heller G. Z. (2010). Ordinal regression models for continuous scales. International Journal of Biostatistics 6, 14. 10.2202/1557-4679.1230 21969972

[kxac048-B30] Marsaglia G. (1963). Expressing the normal distribution with covariance matrix *a* + *b* in terms of one with covariance matrix *a*. Biometrika 50, 535–538.

[kxac048-B31] Maruo K. , Yamaguchi Y., Noma H., Gosho M. (2017). Interpretable inference on the mixed effect model with the Box-Cox transformation. Statistics in Medicine 36, 2420–2434. 28294388 10.1002/sim.7279

[kxac048-B32] Masarotto G. , Varin C. (2012). Gaussian copula marginal regression. Electronic Journal of Statistics 6, 1517–1549.

[kxac048-B33] McGee G. , Stringer A.(2022). Flexible marginal models for dependent data. *Technical Report*, arXiv 2204.07188.

[kxac048-B34] McLain A. C. , Ghosh S. K. (2013). Efficient sieve maximum likelihood estimation of time-transformation models. Journal of Statistical Theory and Practice 7, 285–303.

[kxac048-B35] Molenberghs G. , Verbeke G. (2005). Models for Discrete Longitudinal Data . New York, USA: Springer.

[kxac048-B36] Muff S. , Held L., Keller L. F. (2016). Marginal or conditional regression models for non-normal data? Methods in Ecology and Evolution 7, 1514–1524.

[kxac048-B37] Ogden H. E . (2015). A sequential reduction method for inference in generalized linear mixed models. Electronic Journal of Statistics 9, 135–152.

[kxac048-B38] R Core Team. (2022). R: A Language and Environment for Statistical Computing . Vienna, Austria: R Foundation for Statistical Computing.

[kxac048-B39] Rödel C. , Graeven U., Fietkau R., Hohenberger W., Hothorn T., Arnold D., Hofheinz R.-D., Ghadimi M., Wolff H. A., Lang-Welzenbach M. others. (2015). Oxaliplatin added to fluorouracil-based preoperative chemoradiotherapy and postoperative chemotherapy of locally advanced rectal cancer (the German CAO/ARO/AIO-04 study): final results of the multicentre, open-label, randomised, phase 3 trial. The Lancet Oncology 16, 979–989. 26189067 10.1016/S1470-2045(15)00159-X

[kxac048-B40] Sauter R. , Held L. (2016). Quasi-complete separation in random effects of binary response mixed models. Journal of Statistical Computation and Simulation 86, 2781–2796.

[kxac048-B41] Stroup W. W. (2012). Generalized Linear Mixed Models: Modern Concepts, Methods and Applications . New York, USA: Chapman & Hall/CRC.

[kxac048-B42] Sun T. , Ding Y. (2021). Copula-based semiparametric transformation model for bivariate data under general interval censoring. Biostatistics 22, 315–330. 31506682 10.1093/biostatistics/kxz032

[kxac048-B43] Tamasi B. (2022). *tramME: Transformation Models with Mixed Effects .* R package version 1.0.3. https://CRAN.R-project.org/package=tramME

[kxac048-B44] Tamási B. , Crowther M., Puhan M. A., Steyerberg E., Hothorn T. (2022). Individual participant data meta-analysis with mixed-effects transformation models. Biostatistics 23, 1083–1098. 34969073 10.1093/biostatistics/kxab045 PMC9566326

[kxac048-B45] Tamási B. , Hothorn T. (2021). tramME: mixed-effects transformation models using template model builder. The R Journal 13, 398–418.

[kxac048-B46] Tang N. , Wu Y., Chen D. (2018). Semiparametric Bayesian analysis of transformation linear mixed models. Journal of Multivariate Analysis 166, 225–240.

[kxac048-B47] Thas O. , De Neve J., Clement L., Ottoy J.-P. (2012). Probabilistic index models. Journal of the Royal Statistical Society: Series B (Statistical Methodology*)* 74, 623–671.

[kxac048-B48] van der Vaart A. W. (1998). Asymptotic Statistics . Cambridge, UK: Cambridge University Press.

[kxac048-B49] Varadhan R. (2022). *alabama: Constrained Nonlinear Optimization .* R package version 2022.4-1. https://CRAN.R-project.org/package=alabama

[kxac048-B50] Wang T. , Merkle E. C. (2018). **merDeriv**: derivative computations for linear mixed effects models with application to robust standard errors. Journal of Statistical Software 87, 1–16.

[kxac048-B51] Wang Z. , Louis T. A. (2003). Matching conditional and marginal shapes in binary random intercept models using a bridge distribution function. Biometrika 90, 765–775.

[kxac048-B52] Wu H. , Wang L. (2019). Normal frailty probit model for clustered interval-censored failure time data. Biometrical Journal 61, 827–840. 30838687 10.1002/bimj.201800114

[kxac048-B53] Ypma J. (2013). *SparseGrid: Sparse Grid Integration in R .* R package version 0.8.2. https://CRAN.R-project.org/package=SparseGrid

[kxac048-B54] Zeger S. L. , Liang K.-Y., Albert P. S. (1986). Longitudinal data analysis using generalized linear models. Biometrika 73, 13–22.

[kxac048-B55] Zeger S. L. , Liang K.-Y., Albert P. S. (1988). Models for longitudinal data: a generalized estimating equation approach. Biometrics 44, 1049–1060. 3233245

[kxac048-B56] Zeng D. , Gao F., Lin D. Y. (2017). Maximum likelihood estimation for semiparametric regression models with multivariate interval-censored data. Biometrika 104, 505–525. 29391606 10.1093/biomet/asx029 PMC5787874

[kxac048-B57] Zhang Z. , Charalambous C., Foster P. (2021). A gaussian copula joint model for longitudinal and time-to-event data with random effects. *Technical Report*, arXiv 2112.01941.

