# Correction to: A transformation perspective on marginal and conditional models

**DOI:** 10.1093/biostatistics/kxad017

**Published:** 2023-08-02

Biostatistics, (2022); kxac048, https://doi.org/10.1093/biostatistics/kxac048

In the originally published version of this article, in section *2.1. Joint transformation models* (p.5) paragraph 3, the beginning of the third sentence should read: “Furthermore, F:R → [0,1] is an *a priori* defined cumulative distribution function of some absolute continuous distribution[…]” instead of: “Furthermore, F:R→R is an *a priori* defined cumulative distribution function of some absolute continuous distribution[…]”. In addition, a production error in translating LaTeX content caused some instances of “y” to be presented as “Y” within the paper. These errors have been corrected in the article.

